# Current Intervention Treatments for Food Addiction: A Systematic Review

**DOI:** 10.3390/bs11060080

**Published:** 2021-05-23

**Authors:** Mark Leary, Kirrilly M. Pursey, Antonio Verdejo-Garcia, Tracy L. Burrows

**Affiliations:** 1 School of Health Sciences, College of Medicine, Health and Wellbeing, University of Newcastle, Callaghan, NSW 2308, Australia; mark.leary@uon.edu.au (M.L.); kirrilly.pursey@uon.edu.au (K.M.P.); 2 Priority Research Centre for Physical Activity and Nutrition, University of Newcastle, Callaghan, NSW 2308, Australia; 3 School of Psychological Sciences and Turner Institute for Brain and Mental Health, Monash University, Clayton, VIC 3800, Australia; antonio.verdejo@monash.edu

**Keywords:** food addiction, YFAS, yale food addiction scale, eating addiction, intervention

## Abstract

Research on the concept of food addiction (FA) has steadily grown and, based on a widely used self-report, FA is estimated to affect between 16–20% of the adult population. However, there are few interventions available for people with self-reported FA, and their efficacy is unclear. The primary aim of the review was to examine the efficacy of different interventions, including behavioural/lifestyle, medication and surgical approaches, for reducing symptoms and/or changing diagnosis of FA among adolescents and adults. A secondary aim was to examine the influence of sex as a moderator of intervention effects. A systematic search was performed from 2008–2020 to identify studies that used the YFAS to assess the effectiveness of interventions on FA. Nine studies were identified (*n* = 7 adults, *n* = 2 adolescents) including a total of 812 participants (range 22–256) with an average of 69% females per study. The types of interventions included medications (*n* = 3), lifestyle modification (*n* = 3), surgical (*n* = 2) and behavioural (*n* = 1), with FA being assessed as a secondary outcome in all studies. Five studies in adults reported a significant reduction in FA symptoms or diagnosis from pre to post-intervention, two when compared to a control group and three in the intervention group only. Efficacious interventions included: medication (combination of naltrexone and bupropion, as well as pexacerfont), bariatric surgery and lifestyle modification. No significant changes in FA were reported in adolescent studies. Given few studies were identified by the review, there is insufficient evidence to provide clear recommendations for practice; however, some interventions show potential for reducing self-reported FA outcomes in adults. Future research should explore the longer-term efficacy of interventions and the effectiveness of treatments with sufficient sample sizes.

## 1. Introduction

Food addiction (FA) is characterised by diminished control over the consumption of certain foods (e.g., hyper-palatable energy-dense foods), which persists despite growing negative health consequences [1,2]. Research on the concept of FA has steadily been growing [3] and self-reported FA is estimated to affect between 16–20% of the population [4,5]. FA is often accompanied by symptoms characterised by a sense of loss of control, continued use regardless of adverse consequences and inability to reduce consumption despite the desire to do so [1,6]. Health implications reported to be associated with addictive-like eating behaviours include elevated body mass index (BMI) [4], increased visceral adiposity and links with eating disorders such as binge eating disorder (BED) and bulimia nervosa (BN) [5,7,8], as well as depression, anxiety [5,9], substance-use disorders (SUD) [10] and post-traumatic stress disorder (PTSD) [11].

FA is typically assessed using the Yale Food Addiction Scale (YFAS). Initially developed and validated in 2009 and revised in 2016, the YFAS is a self-report tool designed to assess FA symptoms adopted from the diagnostic and statistical manual for mental disorders (DSM) criteria for substance-use disorders [6,12]. The YFAS provides two scoring options including a FA symptom score and diagnosis [5]. Although the YFAS has the ability to provide a categorisation of FA, it is important to note that the DSM does not recognise FA as a diagnosable condition and, therefore, it is not included in the DSM. The YFAS has been adapted for different populations, is currently available in 13 languages [13] and is suited for an array of research purposes such that there is a suite of tools including shortened versions.

The prevalence of self-perceived FA within the general population has been reported to be as high as 43%, despite many of these individuals not meeting the YFAS criteria for FA [14]. Meadows et al. (2017) reported that participants still exhibited and reported significantly higher levels of problematic eating behaviours, increased dietary restraint, and a reduced sense of control around food than the self-perceived non-FA individuals [14]. Given the high levels of self-perceived FA, there is a need for treatments or management approaches to assist individuals to seek help. However, current interventions are limited, with the majority of available treatments lacking a scientific evidence approach or not utilising health professionals with expertise in behaviour change [15].

The current treatments for FA largely reflect online self-help groups with many groups comprising of large member numbers, such as Overeaters Anonymous (OA), which has an estimated 54,000 members [16]. Previous systematic reviews regarding interventions to treat FA have focused on online support options [15] and psychosocial interventions only [17]. The review of online treatment options by McKenna et al. (n = 13), identified these were predominately self-help groups utilising a 12-step tradition (11 of 13 studies), with a peer-led, spirituality-based format with very few health professionals involved [15]. While a lack of qualified health professionals such as dietitians or psychologists was reported, the review also highlighted the popularity of online approaches for individuals. The review by Cassin et al. (2020) examined eight studies for the effectiveness of psychosocial interventions on FA, such as psychoeducation and intuitive eating [17]. Of the eight studies included in the review, seven were carried out in adults [18,19,20,21,22,23,24] and one in adolescents [25], with all studies having FA as a secondary outcome. While results across all eight studies suggested a reduction in FA outcomes, the included studies used various eating behaviour measurement outcomes, included small sample sizes (n < 100), were generally of poor methodological quality and were likely insufficiently powered to test the impact of the interventions on FA specifically [17].

Whilst both reviews add valuable insights, they only reviewed a limited scope of interventions. Given treatments for FA could be highly varied due to the strong link with other mental health conditions including disordered eating behaviours [5,9,11,26], substance use disorders [10] and increased weight status [4], there are a range of treatments that could be beneficial for those with addictive eating. The scope and classifications of interventions of interest for the current review, therefore, include those interventions that were not reported in previous reviews such as dietary interventions, lifestyle modifications, medication, and surgery.

There is a need to assess emerging evidence from interventions by adopting a broader approach and reviewing other possible treatments that have the potential to improve FA symptoms or assist individuals in behaviour change regarding eating habits. In doing so, this will assist in informing practice for the management of FA, as well as gaining insight into which treatments are being trialled and their effectiveness to extend future research. Furthermore, there are limited studies that have assessed FA within the context of moderators such as the sex of participants. Understanding whether FA treatments are more or less effective for men or women may be beneficial for treatment selection or case management, as previous studies in similar populations such as patients with BED, have found sex differences in the psychological and physiological features that underpin treatment mechanisms [27]. The primary aim of this review was to examine the efficacy of different interventions, including behavioural/lifestyle, medication and surgical approaches, for reducing symptoms and/or changing diagnosis of FA among adolescents and adults. A secondary aim was to examine the influence of sex as a moderator of intervention effects.

## 2. Materials and Methods

A systematic literature search of peer-reviewed studies was performed from 2008–2020 in accordance with the Preferred Reporting Items for Systematic Reviews and Meta-Analysis (PRISMA) statement [28]. We sought to identify studies that used any version of the YFAS to assess the effectiveness of interventions on FA diagnosis or symptom scores in both adults and adolescents. The lower date range for the search criteria was chosen to reflect the emergence and publication of the YFAS tool [29]. The search was conducted in the following databases and based on previous reviews of FA [4,5]: The Cochrane Library, CINAHL (Cumulative Index to Nursing and Allied Health), MEDLINE, EMBASE (Excerpta Medica Database), Scopus, Informit Health Collection, Proquest, Web of Science and PsycINFO. Keywords were informed by previous literature searches. Five sets of search terms were used including terms related to (1) measuring FA, (2) interventions, (3) FA and overeating, (4) eating behaviours, (5) population groups. Specific terms can be seen in Supplementary Materials S1.

### 2.1. Study Selection Criteria

**Figure 1:**
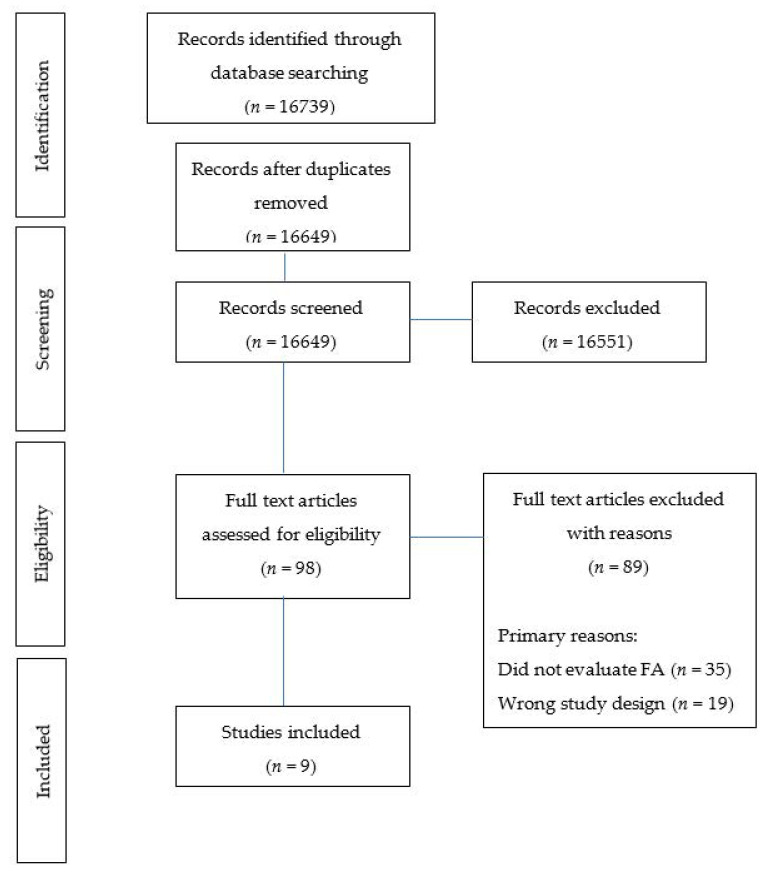
Flow diagram of studies included in the review.

The selection process is shown in Figure 1 (Figure 1 PRISMA flow diagram). Identified studies were uploaded and screened using Covidence [30]. After the removal of duplicates, each title and abstract was screened for inclusion by two independent reviewers with a third reviewer used when discrepancies arose (M.W., M.L., T.B., R.N., K.M.P.). Full texts were then retrieved and screened by two independent reviewers with a third reviewer used when discrepancies arose (M.L., R.N., K.M.P.). If the eligibility of a study’s inclusion was unclear, the article was retrieved for further clarification.

Eligibility criteria for studies to be included in the review were: (1) Participants being adults (18+ years old) and/or adolescent (10–19 years as defined by the World Health Organisation). (2) All validated intervention types were considered, including but not limited to dietary, behavioural, psychological, supplement, medication, and surgical. (3) All comparators considered, including but not limited to, control groups such as waitlist, no control or usual care. (4) Studies needed to report one or more outcomes related to the YFAS tools ((YFAS, modified form of YFAS (m-YFAS, YFAS 2.0 or children YFAS (YFAS-C)) to assess FA; and report either/both the YFAS diagnosis and/or symptom scores as an outcome measure pre- and post-intervention. The FA outcomes reported could either be the primary or secondary outcomes. (5) Study design included but was not limited to randomised control trials (RCT’s) and pre-post studies published in the English language. Review letters to the editor, case studies and conference proceedings were all excluded.

### 2.2. Data Extraction and Synthesis

Data extraction: data were extracted from each of the studies using a standardised extraction form developed by the reviewers and in line with previous reviews. The data extraction form was initially piloted (*n* = 2 studies) and then refined to ensure all the details from each study were retrieved to meet the aims of the review. The main headings included in the data extraction form can be seen in Tables 1 and 2. Data were extracted by one person and checked by an independent reviewer (M.L., R.N.). If data were not available in published studies authors were contacted for additional information (*n* = 2 studies) and where possible pre and post values of YFAS symptom scores and/or diagnosis were determined using t tests.

Data synthesis: Data were synthesised narratively from the standardised data extraction form. Descriptive analysis by subgroups (e.g., population (adults/adolescents), intervention type, sex, age) were included and reported where possible. For the purpose of this review interventions were classified as either: medication, indicating medication was used as part of the intervention; lifestyle modification, which included a combination of dietary with physical activity and/or behavioural modifications as part of the intervention; surgical, indicating a surgical procedure was undertaken as part of the intervention; dietary, where the study included dietary modification as part of the intervention NOT in combination with physical activity or behavioural modification; or behavioural, the study primarily used behavioural modification such as inhibition training, which did NOT include nutrition or physical activity modification. This classification was determined by the authorship team consensus. It should be noted that one study was terminated early [31] due to the United States Federal regulation known as the Common Rule that prohibits the use of deception such as the bogus taste test; however, the study was still included in the review as early termination did not affect the collection of YFAS data contributing to the FA outcomes of the study.

### 2.3. Study Quality

Retrieved studies were assessed by two independent reviewers (M.L., K.M.P.) using the Academy of Nutrition and Dietetics Quality Criteria Checklist [32], as this checklist is a standard tool for the field of nutrition and dietetics and can be used for a broad range of study designs. The quality criteria assessed ten items relating to scientific soundness. The items assessed include the research question, study groups and participants, outcome measures and statistical analysis. Each item was classified as present (“Yes”), absent (“No”), “Unclear” or “Not Available” for each included study. If most (six or more) of the answers to the quality questions were “No”, the study was designated with a negative (−) symbol. If the answers to quality criteria questions 2, 3, 6, and 7 did not indicate that the study was exceptionally strong, the study was designated with a neutral (ø) symbol. If most of the answers to the quality areas were “Yes” (including criteria 2, 3, 6, 7 and at least one additional “Yes”), the study was designated with a positive (+) symbol.

## 3. Results

### 3.1. Search Results

In total, 16649 articles were identified (after duplicates removed) using the search strategy. Following the title and abstract screening, 98 were selected for full-text screening resulting in nine articles that met the inclusion criteria (Figure 1 PRISMA flow diagram). Primary reasons for exclusion included: study objective (i.e., did not evaluate FA, *n* = 35), non-eligible study design (*n* = 19) and not a formal study (i.e., an abstract, *n* = 16).

### 3.2. Description of Included Studies

The characteristics of the included studies are presented in Table 1, and details of the study interventions and outcomes are presented in Table 2. Three studies followed a pre/post-intervention study design with no control group [33,34,35]. Three studies were RCT’s, in two of which the control groups were placebo [31,36] and the other study was an intervention with a food image inhibition training task with the control group receiving no specific instructions or restrictions on viewing images [37]. Three studies included a control group; however, were not randomly assigned [25,38,39]. These control groups included patients that had obesity without BED [38], no treatment [39], or a “Usual Care” method which included a multidisciplinary weight management clinic [25]. The majority of studies were carried out in the USA (*n* = 5), followed by Germany (*n* = 1), Italy (*n* = 1), New Zealand (*n* = 1), and Turkey (*n* = 1).

Of the nine included studies, five used the standard or original YFAS to assess FA [31,33,34,37,39]. Other versions of the YFAS used to assess FA included: children’s YFAS (YFAS-C) in adolescent populations [25,35], an Italian version of YFAS 2.0 [38] and modified YFAS (m-YFAS) [36]. FA was measured as a secondary outcome in all studies across all interventions.

#### 3.2.1. Participants

In total, 812 individuals were included across the nine studies with an average of 90 individuals per study (range 22–256 people). Seven of the nine studies were carried out in adults (>19 years) [31,33,34,36,37,38,39], while two studies were carried out in adolescents (11–18 years) [25,35]. Of the studies that were conducted in adults, the mean age reported was 38.3 years, (range 18–61 years), while the mean age of the adolescent participants was 14.3 years, (range 11–18 years). One study was conducted exclusively in females [37], while all other studies (*n* = 8) included both sexes, with an average of 69% females per study (range 47–93%) [25,31,33,34,35,36,38,39]. The most common population group studied was individuals with overweight/obesity seeking a weight-loss treatment (*n* = 3) [25,33,35]. Other groups included patients undergoing bariatric weight-loss pre- and post-surgery (*n* = 2) [34,39], groups of individuals with disordered eating including BED (*n* = 2) [37,38], a group of individuals with self-reported food cravings (*n* = 1) [31], and a smoking cessation group (*n* = 1) [36]. Six of the nine included studies reported the ethnicity of the study participants, with five of the six studies comprising Caucasian plus one or more other ethnicities [25,31,33,36,39].

#### 3.2.2. Interventions

The type of interventions as categorised for the review in descending order included: “lifestyle modification” (*n* = 3) [25,33,35], “medication” (*n* = 3) [31,36,38], “behavioural” (*n* = 1) [37], “surgical” (*n* = 1) [34] and “surgical and diet” (*n* = 1) [39]. Both adolescent studies were categorised as a “lifestyle modification” intervention type [25,35]. Excluding the two studies that had surgery as the intervention [34,39], the average intervention length was 14.4 weeks (range 2–26 weeks). Follow up post-intervention was reported in three of the nine studies [34,36,39], with an average follow-up duration post-intervention of 45 weeks (range 17–104 weeks). The average reported retention rate at the final follow-up time point was 62.2% (range 30.7–100%).

Five studies reported using a nutrition component/dietary prescription as part of their intervention [25,33,35,38,39]. Of these five studies, one study [33] described the dietary prescription in detail, which consisted of a 10-week calorie-controlled diet of 1000–1200 cal/day (consisting of 4 servings of a chocolate/vanilla liquid shake, which were 160–170 cal each, a pre-packaged/frozen food entrée of 250–300 cal, 1–2 servings of fruit and side salad). The nutrition description of the other four studies was unclear [25,35,38,39]. Four studies incorporated a behavioural component such as behavioural counselling, inhibition training, behaviour change theory or behaviour change goals as part of the intervention [35,37,38]. Four incorporated health professionals as part of their intervention, including psychiatrists (*n* = 1) [38], dietitians or psychologists (*n* = 1) [33], dietitians, physical therapists and psychologists (*n* = 1) [25], and a multidisciplinary team, which did not identify the health professionals involved (*n* = 1) [35].

### 3.3. Outcomes

#### Assessment of Outcomes

Four of the nine studies reported FA diagnosis pre- and post-intervention [33,34,35,36], while eight studies reported FA symptom scores pre- and post-intervention [25,31,33,34,35,37,38,39]. Three studies reported both FA diagnosis and FA symptom scores pre- and post-intervention [33,34,35]. One study reported the endorsement of individual FA symptoms within each of the YFAS diagnostic criteria pre- and post-intervention with no significant differences reported [35]. One study reported FA symptom scores within sex and surgery type categories pre- and post-intervention with no significant differences reported [34].

### 3.4. Effectiveness of Interventions

Seven studies reported an overall reduction in FA diagnosis and/or symptom scores from pre- to post-intervention [25,31,33,34,35,38,39]. Of these seven studies, a statistically significant reduction (*p* < 0.05) was reported in five studies [31,33,34,38,39]. Of the six studies that included a control group [25,31,36,37,38,39], three reported changes in diagnosis or symptom scores that were statistically different from the baseline [31,38,39].

#### 3.4.1. Changes Post-Intervention Diagnosis

Of the four studies that reported FA prevalence pre- and post-intervention [33,34,35,36], one study reported a significant reduction (*p* < 0.05) in FA prevalence from 57.8% at baseline to 7.2% at 6 months and 13.7% at 12 months post-surgical intervention in bariatric surgery patients [34]. Specifically, one study in adolescents reported the pre- and post-intervention prevalence of FA that was 23.1% and 7.7%; however, this was not statistically significant and had a small sample size [35].

#### 3.4.2. Changes Post-Intervention Symptom Scores

Of the eight studies that reported FA symptom scores pre- and post-intervention, five studies, all of which were conducted in adults, reported a statistically significant reduction [31,33,34,38,39]. Two of these five studies, both medication interventions (n = 2) naltrexone and bupropion [38] and pexacerfont [31], reported a significant reduction in FA symptom scores when the intervention group was compared to the control group. While in three studies, a lifestyle modification intervention [33] and two surgical interventions [34,39], reported a significant reduction in FA symptom scores in the intervention group only.

### 3.5. Moderators

#### Changes Post-Intervention Symptom Scores between Sexes and Surgery Type

One study reported the differences in FA symptom scores according to sex and surgery type pre- and post-intervention [34]. While the intervention showed a reduction at a group level, where males had the largest reduction in FA symptom scores compared to females over the same time period, it was not statistically significant at the subgroup level between the sexes. When reported by surgery type, while not statistically significant, results did show an overall reduction in FA symptom scores where the group receiving an omega loop gastric bypass (LGB) surgery reported greater reductions compared to the laparoscopic sleeve gastrectomy (LSG) surgery technique 12 months post-intervention [34].

### 3.6. Quality Assessment/Risk of Bias

Three studies scored a quality rating of positive (+), while six studies scored a quality rating of neutral (ø). No study was scored a quality rating of negative (−). The quality criteria that were missing or not described well for the majority of studies included study participants free from bias (*n* = 7), lack of blinding to prevent bias (*n* = 7) and method of handling withdrawals (*n* = 5). (see Appendix A: Table A1 for quality rating scale)

## 4. Discussion

This review systematically examined the effectiveness of intervention treatments for adolescents and adults with FA as reported by the YFAS tool. Overall, nine studies were identified that have trialled an intervention and reported FA outcomes. Specifically, only one study assessed and reported sex as a moderator on FA outcomes.

Given that few studies were found, there is insufficient evidence to provide clear recommendations for practice. This review found that bariatric surgery, medication and lifestyle modification interventions reported statistically significant reductions in self-reported FA outcomes following interventions, while other intervention types included in this review did not. This is in contrast to the review by Cassin et al. (2020), in which the authors reported there were few effective psychosocial interventions for FA [17]. These differences may be related to differences in the inclusion criteria between the reviews with the current review including a broader array of interventions with some of them more intensive. It is important, however, to distinguish between statistical significance and the clinical meaningfulness of these reported changes for individuals. More studies in this review reported changes in symptom scores than overall FA diagnosis, so this may reflect a change in severity that is deemed important.

Both bariatric surgery studies included in this review, which comprised Roux-en-Y gastric bypass (RYGB) and sleeve gastrectomy (SG) [39] as well as laparoscopic sleeve gastrectomy and omega loop gastric bypass [34], reported significant reductions in FA symptoms over longer time periods (12 months or more). Whereas, both medication studies, which included the combination of naltrexone and bupropion [38] and pexacerfont (a corticotropin-releasing factor (CRF1) antagonist) [31], and the lifestyle modification study which comprised of diet and physical activity [33], reported this significant reduction over a shorter time period (16 weeks or less). Although the limited results prevent recommendations, it is important to note that the studies included in this review used higher-quality designs, and involved health professionals (versus peer-based or self-help) as part of the intervention delivery, in contrast to the majority of the studies included in previous reviews [15,16,17]. However, the majority of the included studies were of neutral quality (n = 6) based on our risk of bias analysis. Of particular note, some studies lacked methodological detail, making replicability difficult. More specifically, FA studies that adopt a nutrition component in their intervention should be encouraged to provide a detailed nutrition description and use checklists such as TIDIER [40] to better ensure progress in the field. Five of the nine included studies reported using a nutrition component/dietary prescription as part of their intervention [25,33,35,38,39]. However, only one study described the dietary prescription in detail [33].

While there were a broad range of interventions investigated and in the context of FA, which often clusters with other chronic disease and comorbidities [4,5,9], it is important to consider the implications for research and practice before treatment options are considered. For example, although bariatric surgery (comprising RYGB, sleeve gastrectomy, laparoscopic sleeve gastrectomy and omega loop gastric bypass) and certain medications (such as naltrexone and bupropion, as well as pexacerfont) may be suitable for those with increased weight status and have been shown to reduce FA symptoms or diagnosis, these treatments would not be applicable for normal-weight participants with FA, and may also have a high financial cost, risks and possible negative side effects for some individuals. Given that a lifestyle modification intervention (incorporating detailed diet and physical activity advice) showed similar reductions in FA symptoms, it may be more practical, cost-effective and safe to adopt this type of treatment, particularly for those with mental health co-morbidities, existing health conditions or a history of other conditions such as trauma or more vulnerable groups such as adolescents where physical change is still occurring.

The results of this review have highlighted a number of methodological limitations that prevent the drawing of strong conclusions on effectiveness. Given FA was a secondary outcome in all nine studies and follow-up duration was limited, it is important to note that the long-term efficacy of these treatments is difficult to determine. Three of the five studies that reported a significant reduction in FA symptom scores occurred in the intervention group only [33,34,39]. Of these three studies, two studies, both of which were bariatric surgery interventions, evaluated the changes in FA greater than 12 months post-intervention [34,39]. In contrast, the majority of studies (n = 5) that reported an overall reduction in FA symptom scores or diagnosis had an intervention length of 6 months or less with no follow up post-intervention [25,31,33,35,38]. In comparison to other interventions such as cognitive behaviour therapy (CBT) for binge eating where the treatment is highly dependent on the individual but usually involves a greater number of sessions, i.e., >20 sessions over a longer follow up [41], intervention lengths of the studies in this review are relatively short. While the main aim of this review was not to assess the long-term efficacy of such FA treatments, previous research has shown FA to be stable over 18 months [42] indicating that these behaviours may take considerable time to achieve change. Therefore, future studies should investigate the longer-term effectiveness of interventions.

The average retention rate of the included studies at the final follow-up time point was low to moderate at 62.2% (range 30.7–100%), when compared to similar FA weight loss studies where retention rates are usually higher with an average of 88% [43,44]. Similarly, when compared to binge eating disorder and cognitive behavioural therapy modalities for eating disorders, where the reported attrition rates range from 3–41% and 22–27%, respectively [45], on average, the retention rates of the included studies are lower. Additional results from the review demonstrate that financial incentives and population group may also affect retention rates, with one study reporting 100% follow up when participants were financially incentivised [25] and three studies reporting low retention rates (less than 50%) for bariatric patients [34,39] and smokers [36].

According to the study by Sevincer et al. (2016), one plausible reason for the low retention rates amongst participants in bariatric surgery studies is that those individuals that were more likely to regain weight post-surgery were less likely to be motivated to maintain contact with the study [34]. This could also be said for other intervention types such as those focused on weight loss, whereby the short-term success of initial weight loss may reflect better compliance with the treatment from participants [46]. Since both bariatric surgical studies also had the longest follow-up time point post-intervention (12 months or more) compared to the other included studies, the likelihood of higher dropouts is therefore not unexpected. Given the identified need for studies with longer follow-up periods, future studies will need to consider mechanisms for retention. Strategies that utilise mobile technologies, such as smartphone apps, are being evaluated [47] and introduced into addictive eating studies [48] and may help improve participant engagement and thus retention rates; however, further research is needed.

There was an overall lack of male participants and analysis of sex differences for changes in FA symptom scores or diagnosis, which is not only limited to this field of research. Eight of the nine included studies comprised largely female participants with an average of 69% females per study [25,31,33,34,35,36,38,39], while one study comprised 100% female participants [37]. Previous FA systematic reviews that reported psychosocial interventions [17], prevalence [4] and associations of FA with mental health [5] also reported the lack of males in FA research. However, this is not surprising given females are more likely to seek help or treatment for disordered eating practices [49]. Nevertheless, given the lack of male representation in FA studies, it is difficult to determine the effectiveness of such interventions between the sexes. Only one of the included studies, which was a bariatric surgical treatment, reported on the differences in FA symptoms within moderators such as sex [34]. While results were not statistically significant between the sexes, males were reported to have a larger reduction in FA symptom scores compared to females over the same time period [34]. Given the underrepresentation of males in FA studies and the possible differences in the effectiveness of treatments between the sexes, future studies may need to identify more effective ways to tailor recruitment messages for both sexes and report the effectiveness of their intervention between sexes.

While a number of studies featured in the systematic review have reported estimates of self-reported FA within adolescent populations to be between 4–38% [5,50], few studies exist that have evaluated the effectiveness of FA treatments within this group. This may be for several reasons, including a vulnerable group with compounding issues of weight management, eating disorder risk and the difficulties in recruiting and retaining individuals in studies such as logistical barriers, cost and motivation [51]. Of the two studies in adolescence that used a lifestyle modification intervention, neither reported a significant reduction in FA post-intervention [25,35]. Adolescence is an important period of neurodevelopment in which the brain can be shaped in response to the context in which one is exposed [35]. Furthermore, adolescence is a time in which obesity and obesity-related problems are more likely to carry into adulthood [35]. Since this life stage is critical for neurodevelopment, and mental health, early identification and treatment of at-risk adolescents may help to prevent or reduce the long-term impact of addictive-like eating behaviours and associated obesity-related complications.

As the number of included studies in this review were limited, with no studies having FA as a primary outcome and the majority of studies assessing FA within a broader parent study, clear recommendations on the most effective treatments were not possible. Despite this inability to draw clear recommendations, there does appear to be some interventions that show potential at reducing FA symptom scores or diagnosis in adults. The strengths of this systematic review include the comprehensive search strategy of multiple databases, completed in accordance with PRISMA reporting guidelines, as well as the inclusion of studies that used the same instrument to measure FA (i.e., YFAS) thus reducing instrument bias. Limitations include that the search was confined to studies reported in English, few studies were identified and there was a lack of diversity in the samples. Studies where the majority of participants are female may not be generalisable to the wider population. Even though the study designs were of a higher quality compared to studies in previous reviews, this review was also limited by the overall quality of the included studies where only three studies had a positive quality rating. Lastly, the limited number of tailored interventions specific to FA retrieved by the search may also have an impact given that FA is not recognised as a diagnosable condition within the DSM.

## 5. Conclusions

There are few treatment studies that report YFAS-based FA symptoms or diagnosis pre- and post-interventions. Although this review did include a broad and diverse range of interventions where the majority of studies reported an overall reduction in FA outcomes, the limited number of studies, the heterogeneity in study design and quality and the differences between endpoints across different intervention modalities make it difficult to provide clear recommendations on the most effective treatments. Overall, bariatric surgery, certain medications, and lifestyle modification interventions appear to show potential for reducing FA symptoms in people with excess weight. However, there are some practical implications that need to be to be considered when deciding on the treatment type such as safety and practicality of the intervention (e.g., the risks associated with surgery or medication versus lifestyle modification) and existing individual comorbidities. Future research is needed to determine the long-term efficacy of interventions on FA outcomes.

## Figures and Tables

**Table 1:** Description of included studies.

Author, Year, Country	Type of Study	Number of Participants (Including Sex)	Retention Rate	Population Studied	Participant Characteristics (Age)	Participant Characteristics (BMI)	Participant Characteristics (Ethnicity)	YFAS Details	Symptom/Diagnosis
Carbone, 2020, Italy [38]	Control Trial Control: Individuals with obesity and non-BED	*n* = 43 Sex: Group 1 (individuals with obesity and BED), F *n* = 17/23 (73.9%), Group 2 (Individuals with obesity and non-BED), F *n* = 10/20 (50%)	79.1% (*n* = 34) Group 1, F *n* = 15/19 Group 2, F *n* = 8/15	Individuals with obesity with/without BED	Group 1 (individuals with obesity and BED) 41.0 ± 13.2 years Group 2 (individuals with obesity and non-BED) 44.4 ± 14.0 years	Baseline BMI: Group 1 (individuals with obesity and BED) 39.0 ± 7.8 kg/m^2^ Group 2 (Individuals with obesity and non-BED) 43.8 ± 9.6 kg/m^2^	Not Reported	YFAS 2.0 Italian version	Symptom
Chao, 2019, USA [33]	Pre/Post No control	*n* = 178 Sex: F *n* = 156 (87.6%)	77.5% (*n* = 138)	Individuals that are OW/OB seeking WL	44.2 ± 11.2 years	Baseline BMI: 40.9 ± 5.9 kg/m^2^	Black, White, other	YFAS (original)	Symptom + Diagnosis
Epstein, 2016, USA [31]	RCT Control: placebo (medication unknown)	*n* = 31 Sex: Treatment group, F *n* = 10/13 (76.9%), Placebo group, F *n* = 15/18 (83.3%)	74.2% (*n* = 23) Treatment group, *n* = 10/13Placebo group, *n* = 13/18	Adults that scored ≥15 DRS for food craving	Treatment group 30.8 ± 8.3 years Placebo group 32.8 ± 10.7 years	Baseline BMI: Treatment group 33.0 ± 11.4 kg/m^2^ Placebo group 36.4 ± 8.3 kg/m^2^	African American, European American, other	YFAS (original) recorded daily	Symptom
Giel, 2017, Germany [37]	RCT Control: no restrictions, control condition (CC group)	*n* = 22 Sex: F *n* = 22 (100%)	90.1% (*n* = 20)	Patients diagnosed with BED	36.6 ± 11.9 years	BMI: 29.6 ± 6.3 kg/m^2^	Not Reported	YFAS (original) German version	Symptom
Murray, 2019, USA [39]	Control Trial Control: (no Treatment)	*n* = 55 Sex: F *n* = 25 (93%)	49.1% (*n* = 27) Surgery *n* = 16 Diet *n* = 6 No treatment *n* =5	Patients undergoing Bariatric Surgery	32.7 ± 7.6 years	BMI: 44.3 ± 4.4 kg/m^2^	Hispanic/Latino, Black/African American, White, Native American, Pacific Islander, other	YFAS (original)	Symptom + Diagnosis
Nordin, 2017, New Zealand [36]	RCT Control: placebo (oral spray unknown)	*n* = 256 Sex: F *n* = 140 (54.7%)	54.7% (*n* = 140) attended at least 1 of the F/up visits, 48.4% (*n* = 124) attended at least 1 of the 1 or 3 month F/up visits, 36.7% (*n* = 94) attended at least 1 of the 6 or 12 month F/up visits	Adult smokers wishing to quit	46.2 ± 12.2 years	BMI: 27.4 ± 6.2 kg/m^2^ (range 16.4–74.1 kg/m^2^)	Caucasian, Maori, other	YFAS (modified version)	Diagnosis
Sevincer, 2016, Turkey [34]	Pre/Post No control	*n* = 166 Sex: F *n* = 128 (77.1%)	50% (*n* = 83) at 6 months, 30.7% (*n* = 51) at 12 months	Patients undergoing Bariatric Surgery	35.6 ± 9.8 years	BMI pre-surgery: 47.0 ± 7.1 kg/m^2^ (range 36.4–69.4 kg/m^2^)	Not Reported	YFAS (original) Turkey version	Symptom + Diagnosis
Tompkins, 2017, USA [35]	Pre/Post No control	*n* = 26 Sex: F *n* = 14 (53.8%)	50% (*n* = 13), F *n* = 6	Adolescents that are OW/OB seeking WL	Age: 14.0 ± 1.9 years (range 11–18 years)	BMI: 33.0 ± 6.3 kg/m^2^ (range 24.3–47.3 kg/m^2^)	Caucasian	YFAS (children version)	Symptom + Diagnosis
Vidmar, 2019, USA [25]	Control Trial Control: Usual care (multidisciplinary weight management clinic = Empower group)	*n* = 35 Sex: Empower group, F *n* = 8/17 (47.1%) Application group, F *n* = 13/18 (72.2%)	Empower group 35% (*n* = 6) at 6 months Application group 100% (*n* = 18) at 6 months	Adolescents that are obese seeking WL	Empower group 14.4 ± 1.8 years Application group 14.4 ± 1.7 years	BMI: Not Reported	Ethnicity: Hispanic, Caucasian, Black, other	YFAS (children version)	Symptom

BED, binge eating disorder; BMI, body mass index; DRS, dietary restraint scale; F/up, follow up; Ix, intervention; OW/OB, overweight/obese; RCT, randomised control trial; WL, weight loss; YFAS, yale food addiction scale.

**Table 2:** Outcomes of included studies.

Author, Year, Country	Intervention Type	Prevalence of FA as per YFAS Diagnosis	YFAS Symptoms, Mean (SD) Pre Intervention	YFAS Symptoms, Mean (SD) Post-Intervention	Intervention Length	Follow Up (Post-Intervention)	Quality Rating
Carbone, 2020, Italy [38]	**Medication**: naltrexone + bupropion + **Lifestyle modification**: hypocaloric diet reducing daily cals of about 500 cal, behavioural counselling, physical activity	Not Reported	Group 1 (individuals with obesity and BED)—6.5 (3.5) (*n* = 23) Group 2 (individuals with obesity and non-BED)—3.4 (2.5) (*n* = 20)	Group 1 (individuals with obesity and BED) *n* = 19, 3.4 (3.6) Group 2 (individuals with obesity and non-BED) *n* = 15, 2.9 (3.0)	16 weeks	Nil	Neutral
Chao, 2019, USA [33]	**Lifestyle modification**: 14 × 90 min lifestyle mod sessions led by registered dietitians or psychologists. Weeks 2–12 follow 1000–1200 cal/day diet (4 serves of choc/vanilla liquid shakes 160–170cal/shake, a pre-packaged/frozen food entrée 250–300 cal, 1–2 serve fruit and side salad. weeks 12–14 refeeding diet replacing shakes with conventional foods. week 6 > increase physical activity to reach 175 min/week by week 14	FA diagnosis: baseline = 6.7% (*n =* 12) of 178 participants post = 1.4% (*n* = 2) of 138 participants	2.24 (1.58) (*n* = 138)	1.93 (1.24) from *n* = 138	14 weeks	Nil	Positive
Epstein, 2016, USA [31]	**Medication**: pexacerfont- corticotropin-releasing factor (CRF) antagonist vs placebo medication (unknown)	Not Reported	Treatment group 6.5 (4.3) (*n* = 13) Placebo group 7.8 (4.2) (*n* = 18) * Treatment group 2.4 (2.6) Placebo group 4.1 (2.3)	Reported as least-squares means: pexacerfont, 1.59 ± 0.30; placebo, 2.49 ± 0.27 * Treatment group 1.9 (2.9) Placebo group 2.0 (3.9)	35 days	Nil	Positive
Giel, 2017, Germany [37]	**Behavioural**: food specific inhibition training	Not Reported	FIT group—3.4 (1.8) (*n* = 10) CC group—3.4 (1.4) (*n* = 10)	FIT group *n* = 10 3.4 (1.3) CC group *n* =10 3.5 (1.8)	2 weeks	4 weeks post-intervention Binge eating only, no YFAS	Positive
Murray, 2019, USA [39]	**Surgical**: (RYGB + SG) + **Diet**: weight loss (liquid meal replacement diet for 3 months) or no treatment (control)	FA diagnosis: baseline = 6.3% surgery group, 33.3% diet group, 40% no treatment group	Baseline surgery 1.9 (*n* = 16) diet 2.7 (*n* = 6) no treatment 3.2 (*n* = 5) (interpreted from graph)	4 months Surgery 1.2 (*n* = 16) Diet 1.6 (*n* = 6) No treatment 3.5 (*n* = 5) 24 months Surgery 0.9 (*n* = 16) Diet 2.3 (*n* = 6) No treatment 2.8 (*n* = 5) (interpreted from graph) Sig diff between baseline and both f/up time points in surgery group only	Surgery	4 months + 24 months	Neutral
Nordin, 2017, New Zealand [36]	**Medication**: oral nicotine spray vs oral placebo spray (unknown)	FA diagnosis: baseline = 0.8% (*n* = 2) 1 and 3 month = 0% 6 and 12 month = 1.1% (*n* = 1)	Not Reported	Not Reported	6 months	1 and 3 months (early F/up) 6 and 12 months (late F/up)	Neutral
Sevincer, 2016, Turkey [34]	**Surgical**: laparoscopic sleeve gastrectomy + omega loop gastric bypass	FA diagnosis: baseline = 57.8% (*n* = 96) 6 month = 7.2% (*n* = 6) 12 month = 13.7% (*n* = 7)	3.75 (1.44) (*n* = 166)	6 month 2.79 (1.00) (*n* = 83) 12 month 2.96 (1.25) (*n* = 51)	Surgery	6 months + 12 months	Neutral
Tompkins, 2017, USA [35]	L**ifestyle modification**: 12 week multidisciplinary weight management program (consisting of physical activity and nutrition instruction, as well as behavioural instruction derived from SCT) in outpatient setting	FA diagnosis: Baseline = 30.7% (*n* = 8) FA diagnosis completers (*n* = 13) pre 23.1% (*n* = 3/13), post 7.7% (*n* = 1/13)	2.35 (1.8) (*n* = 26) 2.08 (1.6) (*n* = 13) (completers)	1.00 (0.9) (*n* = 13) (completers)	12 weeks	Nil	Neutral
Vidmar, 2019, USA [25]	**Lifestyle modification**: mobile-health technology app (mHealth). *Empowergroup—*consists of a team of physicians, dietitians, physical therapists and psychologists. Individual behaviour change goals for healthy eating, physical activity, emotional well-being and family support F/up at monthly visits. *Applicationgroup—*2 × clinic visits at 2 + 6-month intervals + ongoing support via txt msg + weekly phone calls. *Stage* *1—*Participants withdrew from 2 self-selected problem foods at a time, with goal of total abstinence for min 10 consecutive days. *Stage 2—*Eliminating snacking between meals. *Stage 3—*Excessive food amounts reduced through weighing and recording serves into the application	Not Reported	N=10/18 (55%) of Application group scored 4 or more on YFAS (children version) at baseline * 4.22 (1.35)	Reported as: no linear relationship between the change in zBMI and YFAS (children version) at baseline (coef = 0.01, 95%CI = −0.02, 0.04. *p* = 0.52) 17% (3/18) had negative YFAS (children version) scores upon completion of intervention * 3.78 (1.48)	6 months	Nil	Neutral

CC, control condition; CI, confidence interval; FA, food addiction; FIT, food inhibition training; F/up, follow up; RCT, randomised control trial; RYGB, Roux-en-Y gastric bypass; SCT, social cognitive theory; SD, standard deviation; SEM, standard error mean; SG, sleeve gastrectomy; YFAS, yale food addiction scale; zBMI, body mass index Z-score. * additional data provided by the author of the study.

## Data Availability

Futher details about this study can be found at https://osf.io/g98hp or within the Supplementary Materials search protocol.

## Supplementary Materials

The following are available online at https://www.mdpi.com/article/10.3390/bs11060080/s1. Systematic review search protocol.

## Appendix A

**Table A1:** Assessment of study quality using the Academy of Nutrition and Dietetics Quality Criteria Checklist [32].

Study (1st Author, Year)	1. Was the Research Question Clearly Stated?	2. Was the Sample of Study Participants Free from Bias?	3. Were Study Groups Comparable?	4. Was Method of Handling Withdrawals Described?	5. Was Blinding Used to Prevent Introduction of Bias?	6. Were Intervention/Therapeutic Regimens/Exposure Factor or Procedure and Any Comparisons Described in Detail?	7. Were Outcomes Clearly Defined and the Measurement Valid and Reliable?	8. Was the Statistical Analysis Appropriate?	9. Were Conclusions Supported by Results with Biases and Limitations Considered?	10. Is Bias Due to Study’s Funding or Sponsorship Unlikely?	Overall Quality (+, ø, −)
**Carbone E et al., 2020 [38]**	Y	N	Y	Y	N	Y	Y	Y	Y	Y	ø
**Chao A, et al. 2017 [33]**	Y	Y	N/A	Y	N	Y	Y	Y	Y	Y	+
**Epstein D et al., 2016 [31]**	Y	U/C	Y	N	Y	Y	Y	Y	Y	Y	+
**Giel K et al., 2017 [37]**	Y	U/C	Y	Y	Y	Y	Y	Y	Y	U/C	+
**Murray S et al., 2019 [39]**	Y	N	Y	Y	U/C	N	Y	Y	Y	Y	ø
**Nordin A et al., 2017 [36]**	Y	U/C	U/C	N	N	N	Y	Y	Y	Y	ø
**Sevincer G et al., 2016 [34]**	Y	N/A	Y	N	N	Y	Y	Y	Y	Y	ø
**Tompkins C et al., 2017 [35]**	Y	N	N/A	N	N	N	Y	Y	Y	Y	ø
**Vidmar A et al., 2019 [25]**	Y	N	Y	N	N	Y	Y	Y	Y	Y	ø

Y: Yes; N: No; U/C: Unclear; N/A: Not applicable; (+): positive quality study; (ø): neutral quality study; (−): negative quality study.
