# Supplementary material for: Current Intervention Treatments for Food Addiction: A Systematic Review
Source: Behav Sci (Basel). 2021 May 23;11(6):80. doi: 10.3390/bs11060080 (PMC8224570; doi:10.3390/bs11060080)
Supplement: Supplementary file 1 [file behavsci-11-00080-s001.zip › behavsci-1186232-supplementary.pdf]

<u>YFAS Systematic Review Search Protocol</u>

Key words

Set 1

- Yale food addiction scale
- YFAS
- questionnaire
- scale
- instrument
- measure
- tool

Set 2

- treatment
- therapy
- intervention
- program

Set 3

- feeding and eating disorders
- compulsive eat
- overeat
- hyperphagia
- eating addiction
- food addiction
- hedonic eat
- addictive eat
- craving
- binge eat

Set 4

- food preferences
- food habits
- food
- eat behaviour
- eat
- feeding behaviour
- addictive behaviour
- behaviour addiction
- substance-related disorders
- substance-use disorders

Set 5

- teen
- adolescent
- adult
- paediatrics
- patient
- participant

- Combine set 1 and set 2 and set 3 and set 4 and set 5

Databases

- The Cochrane Library,

- CINAHL (Cumulative Index to Nursing and Allied Health)
- MEDLINE
- EMBASE (Excerpta Medica Database)
- Scopus
- Informit Health Collection
- Proquest
- Web of Science
- PsycINFO

Limits: humans, English language, 2008 onwards

Medline, Embase, PsychInfo, CINAHL

(yale food addiction scale or YFAS).mp.
(scale* or instrument* or measure* or questionnaire* or tool*).mp.
1 or 2
treatment*.mp.
therap*.mp.
(intervention* or program*).mp.
4 or 5 or 6
"feeding and eating disorders"/
"compulsive eat*".mp.
overeat*.mp.
Hyperphagia/
eating addict*.mp.
food addiction/ or food addict*.mp.
"hedonic eat*".mp.
"addictive eat*".mp.
Craving/
"binge eat*".mp.
8 or 9 or 10 or 11 or 12 or 13 or 14 or 15 or 16 or 17
Food Preferences/
"food habits".mp.
Food/
(eat* behaviour or eat* behavior or eat*).mp.
Feeding behavior/ or feed* behaviour.mp.
Behavior, Addictive/ or behave* addiction.mp.
Substance-Related Disorders/
substance-use disorder.mp.
19 or 20 or 21 or 22 or 23 or 24 or 25 or 26
teen*.mp. or adolescent/ or adolesc*.mp.
adult/ or adult*.mp.
Pediatrics/ or paediatrics.mp.
Patients/ or patient*.mp. or participant*.mp.
28 or 29 or 30 or 31
Combine 3 and 7 and 18 and 27 and 32

Cochrane, Scopus, Web of Science, Proquest

("Yale food addiction scale" OR YFAS OR questionnaire* OR scale* OR instrument* OR measure* OR tool*) AND (treatment* OR therap* OR intervention* OR program*) AND ((feeding and "eating disorders") OR "compulsive eat*" OR overeat* OR hyperphagia OR "eating addict*" OR ("food addiction" OR "food addict*") OR "hedonic eat*" OR "addictive eat*" OR craving OR "binge eat*") AND ("food preferences" OR "food habits" OR food OR ("eat* behaviour" OR "eat* behavior" OR eat*) OR ("feed* behaviour" OR "feed* behavior") OR ("addictive behaviour" OR "behave* addiction") OR "substance-related disorders" OR "substance-use disorders") AND (teen* OR adolescent*) OR (adult OR adult*) OR (paediatrics OR pediatrics)

OR (patient* OR participant*)
